# Determination of the Human Antibody Response to the Neutralization Epitopes Encompassing Amino Acids 313–327 and 432–443 of Hepatitis C Virus E1E2 Glycoproteins

**DOI:** 10.1371/journal.pone.0066872

**Published:** 2013-06-24

**Authors:** Ruyu Liu, Huiying Rao, Jianghua Wang, Xingwang Xie, Dong Jiang, Xiaoben Pan, Ping Zhao, Henghui Zhang, Lai Wei

**Affiliations:** 1 Peking University People’s Hospital, Peking University HepatologyInstitute, Beijing, China; 2 Beijing Key Laboratory of Hepatitis C and Immunotherapy for Liver Diseases, Beijing, China; 3 Department of Microbiology, Shanghai Key Laboratory of Medical Biodefense, Second Military Medical University, Shanghai, China; Institut Pasteur, France

## Abstract

It has been reported that monoclonal antibodies (MAbs) to the E1E2 glycoproteins may have the potential to prevent hepatitis C virus (HCV) infection. The protective epitopes targeted by these MAbs have been mapped to the regionsencompassing amino acids 313–327 and 432–443. In this study, we synthesized these two peptides and tested the reactivity of serum samples from 336 patients, 210 of whichwere from Chronic Hepatitis C (CHC) patients infected with diverse HCV genotypes.The remaining 126 samples were isolated from patients who had spontaneously clearedHCV infection.In the chronic HCV-infected group (CHC group), the prevalence of human serum antibodies reactive to epitopes 313–327 and 432–443was 24.29%(51 of 210) and4.76%(10 of 210),respectively. In thespontaneousclearance group (SC group),the prevalence was 0.79%(1 of 126) and 12.70%(16 of 126), respectively.The positive serum samples that contained antibodies reactive to epitope 313–327 neutralizedHCV pseudoparticles (HCVpp) bearing the envelope glycoproteins of genotypes 1a or 1b and/or 4, but genotypes 2a, 3a, 5 and 6 were not neutralized. The neutralizing activity of these serum samples could not be inhibited by peptide 313–327. Six samples (SC17, SC38, SC86, SC92, CHC75 and CHC198) containing antibodies reactive to epitope 432–443 had cross-genotype neutralizing activities. Theneutralizing activityof SC38, SC86, SC92 and CHC75waspartiallyinhibited by peptide 432–443. However,the neutralizing activity of sample SC17 for genotype 4HCVpp and sample CHC198 for genotype 1b HCVppwere notinhibited by the peptide.This study identifies the neutralizing ability of endogenous anti-HCV antibodies and warrants the exploration of antibodies reactive to epitope432–443as sources for future antibody therapies.

## Introduction

Worldwide, an estimated 130–200 million people are infected withHCV[1]–[4]. Among these individuals,approximately 80% of the infections will progress to chronic hepatitis C, whichcan lead to liver cirrhosis and hepatocellular carcinoma [5], [6]. Currently, there is no available vaccine to prevent HCV infection, and polyethylene glycol interferon-α-based standard anti-virus treatment isless efficacious against the most common genotypes 1 and 4 [7]. Thus, there is an urgent need for the development of an effective vaccine and new therapeutic regimens.

HCV variants are classified into 6 genotypes and more than 90 subtypes [8], [9]. Adding to the complexity, the virus of an infected individual may have extensive heterogeneity and exist as a quasispecies, which enables thevirus to effectively evade host immunity. When viral clearance is successful, some reports have shown this process to be associated with hostgenetic backgrounds including host HLA types, cytokine andchemokine expression (e.g., IL-10, IL-28B, and CCR5)[10]–[15].Moreover, several studies indicate that a strong, multi-specific, and long-lasting cellular immune response is important for the control of viral infection in acute hepatitis C[16]–[18].

Neutralizing antibodies also play an important role in controlling HCV infection. Studies have suggested that viral clearance is associated with a rapid induction of neutralizing antibodies in the early phase of infection [19], [20], and a large collection of antibodies has been reported to prevent HCV pseudoparticles (HCVpp) or Cell culture-produced HCV (HCVcc) infection[9], [21]–[29]. One other antibody, named D32.10, plays a protective role by inhibiting the interaction between serum-derived envelope HCV particles and hepatocytes [30], [31].

Among these protective antibodies, two monoclonal antibodies (MAbs), which recognize an epitope including amino acid residues 313 to 327 of glycoprotein E1,wererecently reported to strongly neutralize diverse genotypes of HCVpp (1a, 1b, 4, 5 and 6) and to a lesser extent genotype 2a HCVpp [24].The report suggests thatMAbs to the 313–327 region of glycoprotein E1 may have the potential to prevent HCV infection.MAbs specific amino acids 432–443 of glycoprotein E2 can also neutralize genotypes 1a and 1b [32], [33].The MAbs to an overlapping epitope 434–446 can neutralize 1a, 2a, 4, 5 and 6 HCVcc [28]. The ability of anti-sera specific for the epitope spanning 432–443 to inhibit entry of HCVpp into Huh-7 cells was tested. Study shows that these anti-sera can prevent HCVpp bearing the envelope glycoprotein H77c from entering the cell [34]. These findings may be useful for the development of novel immunotherapeuticstrategies and prophylactic vaccines against HCV.However, the described antibodies or anti-sera were discovered either in animal models [34], [35]or in one single HCV infected patient [24]. Thus, confirming theirneutralizingactivitiesusinglarge size human serum samples of HCV-infected individualsarenecessary.

In this study, the reactivity of serum samples from 336 HCV-infected individuals was tested against peptide 313–327 and peptide 432–443. HCVpp and HCVccneutralization and peptide-blocking assayswere then used to test the neutralizing activity of the positive serum samples.Finally, we determined the prevalence of these two epitopes-reactive antibodies and their cross-genotype neutralizing activities. This study confirmed that epitope 432–443 reactive antibodies have cross-genotype neutralizing activities.

## Materials and Methods

### Patient Samples

Serum samples were obtained from 336 HCV antibody-positive subjects (Table 1), and tested by Anti-HCVVITROS Immunodiagnostic Products (Ortho, Wales, UK). Chronic Hepatitis C patients represented 210 of these serum samples (group 1, CHC group). The remaining 126 samples were from individuals who had spontaneously cleared the HCV infection (anti-HCV positive, RNA-negative) (group 2, spontaneous clearance group, SC group).The Ethical Committee of Human Experimentation in Peking University People's Hospital approved the study. Informed consent for the experimental use of serum samples was obtained from all patients in written form according to the hospital's ethical guidelines. Sera complement was inactivated by heating to 56°C for 30 min. All serum samples were stored at −80°C upon collection. The control group was composed of 60 normalhuman serum (NHS) samples from blood donors, which were negative for HCV, human immunodeficiency virus (HIV), andhepatitis B virus (HBV).

**Table 1:** Demographic characteristics of the 336 subjects.

State	Chronic infection	Self-limited infection
Age(M±SD)	50.87±5.77	52.31±6.6
Sex (Male/Female)	104/106	60/66
Genotypes (1/2/1 mixed 2)*	126/67/17	ND
Anti-HCV(S/CO, M±SD)#	29.62±3.40	22.65±8.11
HCV RNA⋇(M±SD)	5.24E+06±9.29E+05	Target not detected

* HCV genotype determined by Lipa (Siemens Healthcare).

#HCV Abs detected by ELISA (S/Co, Abbott).

⋇ Viral loads were quantified through quantitative real-time Taqman PCR (IU/mL, Roche).

ND = not determined.

SD = standard deviations.

### Determination of the Infecting HCV Genotypes

A total of 210 serum samples from chronically infected individuals were tested using the VERSANT HCV Genotype 2.0 Assay(Siemens Healthcare, Belgium) according to the manufacturer’s instructions.

### Peptides

The biotinylated peptides 313–327 (representative of the region encompassing aa 313–327 of the HCV H77 polyprotein) of the E1 glycoprotein and 432–443 of E2were synthesized as follows: Bio-ITGHRMAWDMMMNWS-amide(313–327), Bio-SLNTGWLAGLFY-amide(432–443) (Invitrogen, Shanghai, China), andwere resuspended in dimethyl sulfoxide (2.5% final), diluted with phosphate-buffered saline (PBS) to 1 mg/ml, and stored at −20°C. We also synthesized two peptides as positive and negative controls as previously described byTarr et al. [9].The positive control peptide contained a non-structural protein 4 (NS4) immunogenic epitope (Bio-KPAIIPDREVLYREFDEM-amide; aa 1691–1708) [36] and the negative control peptide corresponds to a sero-reactive region of the rabies virus glycoprotein (Bio-VNLHDFRSDEIE-amide) [37].

### Epitope-reactive Antibodydetection

Anti-peptide 313–327 and 432–443 antibodies were detected usingindirect ELISA as described previously [31]. Briefly, 100 µl of streptavidin (Promega, Madison, WI, USA) wascoated onto 96-well Maxisorpmicrotiter plates (Nunc, Roskilde, Denmark) by incubation (1 mg/ml stored solution diluted 1/100 in 0.05 M carbonate buffer [pH 9.6, Sigma, Louis, Mo, USA], i.e., 10 µg/ml final concentration) in each well (1 µg/well) overnight at 4°C. Plates were washed three times with 300 µL ofPBS per well. The wells were blocked with 200 µL of PBS 1×containing 10% goat serum (Gibco/Invitrogen, Grand Island, NY, USA) for 1 hour at 37°C. Plates were washed three times with PBS, and 100 µL of the biotinylated peptide solution (10 µg/mL) was added to each well for 2hours at 37°C. After another wash with PBS, 100 µL of human serum, diluted 1/250 in PBSTG (PBS containing 0.05% Tween 20 and 10% goat serum), was added to the wells and incubated for 2 hours at 37°C. The plates were washed four times with 300 µL ofPBST per well.Peroxidase-conjugated goat anti-human immunoglobulin G (IgG) (Sigma, Louis, Mo, USA) was diluted 1/5000 in PBSTG and added to each well for 1 hour at 37°C. The plates were washed four times with 300 µL ofPBST per well. Then, the substrate (o-phenylenediaminedihydrochloride and H_2_O_2_[Sigma, Louis, Mo, USA]) was added, and after 30 minutes, 100 µL of 2 N HCl was added to each well to stop the reaction. Optical density (OD) valueswere measured at 490nm using an ELISA plate reader. Each plate contained 10 NHS control wells. The cutoff value for the peptide was calculated as the mean value obtained from at least 10 NHS+3SD.

### HCVpp Production

HCVpp were produced by co-transfection of 293T cells with an HCV envelope protein expression vector and a packaged plasmid based on the HIV-1 strain NL4–3 (Invitrogen) as described previously [32], [38]. Briefly, 293T cells were co-transfected with expression plasmids encoding the HCV envelope glycoproteins, HIV gag/pol (pLP1), HIV rev (pLP2), and pLenti7 encoding Emerald Green Fluorescent Protein (EmGFP) [39]. HCV envelope expression plasmids used here include genotype 1a strain H77 (provided by F. L. Cosset, INSERM U758, Lyon, France), genotype 1b strain Con-1 (provided by C. Rice, Rockefeller University, New York, NY), and genotypes 2a(clone UKN2A1.2), 3a(clone UKN3A1.28C), 4(clone UKN4.21.16),5(UKN5.14.4) and 6(UKN6.5.340)(provided by J. K. Ball, The University of Nottingham, United Kingdom). After 48 hours of co-transfection, the virus-containing supernatants were harvested, filtered through 0.45 µm membranes; concentrated 20fold (Pall, Macrosep Advance Centrifugal Device, 100K, USA) and used to infect Huh7.5 cells.

### HCVpp Neutralization Assays

Huh7.5 cells were pre-seeded into 96-wells platesat a density of 1×10^4^ per well. The next day, the HCVpp supernatants (20 µl/well) were incubated with each positive sera andcontrol sera at various concentrations, plus 4 µg/ml polybrene at 37°C for 1 hour. The mixtures were then added to each well. After incubation at 37°C for 5hours, the supernatants were replaced with fresh complete medium and incubated for 72 hours at 37°C. HCV entry was determined as the percentage of GFP-positive cells measured by flow cytometric analysis. Serum-mediated neutralization was described as the concentration thatinhibited infection of HCVpp derived from diverse genotypes by 50%.

### HCVpp Neutralization Peptide-blocking Assays

Huh7.5 cells were pre-seededinto 96-well platesat a density of 1×10^4^ per well. The next day, virus-containing supernatants were incubated with each positive serum and control serum sample at a dilution that was approximately the 50% inhibiting concentration (IC50) value.The peptides 313–327/432–443 or an irrelevant negative control peptide plus 4 µg/ml polybrene were added and incubated at 37°C for 1 hour. A peptide concentration of 10 µg/ml was used for inhibition. The peptide mixtures were added to each well. After incubation at 37°C for 5 hours, the supernatants were replaced with fresh complete medium and incubated for 72 hours at 37°C. HCV entry was determined as the percentage of GFP-positive cells measured by flow cytometric analysis.

### Cell Culture-produced HCV (HCVcc) Generation

Cell culture supernatant was collected from full-lengthJFH-1 and J6/JFH-1 RNA-transfected Huh7.5 cells and was used to infect Huh7.5 cells grown in T25 flasks at a multiplicity ofinfection (MOI) of 0.01. The infected cells were passagedat 3-day intervals with 1∶3split ratios intoprogressively larger culture vessels. At 12 days post-infection, Viral stocks were obtained by harvesting cell culture supernatantsclarifiedby centrifugation (5 min at 4000 rpm), and stored in aliquots at −80°C.

### HCVcc Neutralization Assays

Huh7.5 cells were seeded in 96-well plates 1 day before infection at a density of 7×10^3^ per well.HCVcc was incubated with each positive sera andcontrol sera at various concentrationsfor 1 hour at 37°C. The mixture was then incubated with Huh7.5 cells for 5 hours at 37°C, and cultured for 72hours after the addition of fresh medium. Each test was performed in triplicate. Neutralization activity of the sera was evaluatedby counting of HCV NS3-postive foci.Serum-mediated neutralization was described as the dilution thatinhibited infection of HCVcc derived from JFH-1 and J6/JFH-1. The percentage of neutralization was estimated by comparison with the mean neutralization for triplicate HCVcc incubations with an irrelevant-antibody control.

### HCVcc Neutralization Peptide-blocking Assays

Huh7.5 cells were pre-seededinto 96-well plates 1 day before infectionat a density of 7×10^3^ per well. HCVcc was incubated with each positive and control serum sample at a dilution that was approximately the 50%inhibiting concentration (IC50) value.The peptides 313–327/432–443 or an irrelevant negative control peptide wereadded and incubated at 37°C for 1 hour. A peptide concentration of 10 µg/ml was used for inhibition. The peptide mixtures were added to each well. After incubation at 37°C for 5 hours, the supernatants were replaced with fresh complete medium and incubated for 72 hours at 37°C. Each test was performed in triplicate. Neutralization activity of the sera was evaluatedby counting of HCV NS3-postive foci. The percentageof neutralization was evaluated by comparison with the mean neutralizationfor triplicate HCVcc incubations with an irrelevant-antibody control.

### Indirect Immunofluorescence Staining

Indirect immunofluorescence staining was performed as previously described [40]. Briefly, Huh7.5 cells were seeded on 96 well plates (7000 cells/well). After 5 hours of incubation at 37°C, the supernatants were replaced with fresh complete medium. Following an additional 72-hour incubation, the cells were fixed in 4% paraformaldehyde for 30 min at room temperature (RT), and then blocked for 60 min in a blocking buffer (3% BSA, 0.3% Triton X-100, 10% FBS in PBS). Following this, the cells were incubated with an anti-hepatitis C virus NS3 antibody (Abcam, ab13830) at a 1∶200 dilution. After 2 hours of incubation at RT, cells were washed extensively with PBS and then incubated with Alexa Fluor 488 rabbit anti-mouse IgG (Invitrogen, A11059) at a 1∶200 dilution for 1 hour. Following PBS washes, the numbers of fluorescent foci(defined as a cluster of infected cells immunostained positive for NS3 antigen) per well were counted.

### Statistical Analysis

Statistical comparison of the prevalence of epitope-reactive antibodies between two groups of patients was performed with a*χ* ^2^ test. Statistical comparison of HCVpp and HCVccneutralization assay results and peptide-blocking assay results betweengroups was performed with One-Way ANOVA analysis, and *P* values were calculated using the SPSS 16.0 software. *P* values corresponding to <0.01 and, 0.01<*P*<0.05 are represented by ** and * respectively. *P*values >0.05 were not considered significant and left un-denoted.

## Results

### Determination of the cutoff value for epitope-reactiveantibody

**Figure 1:**
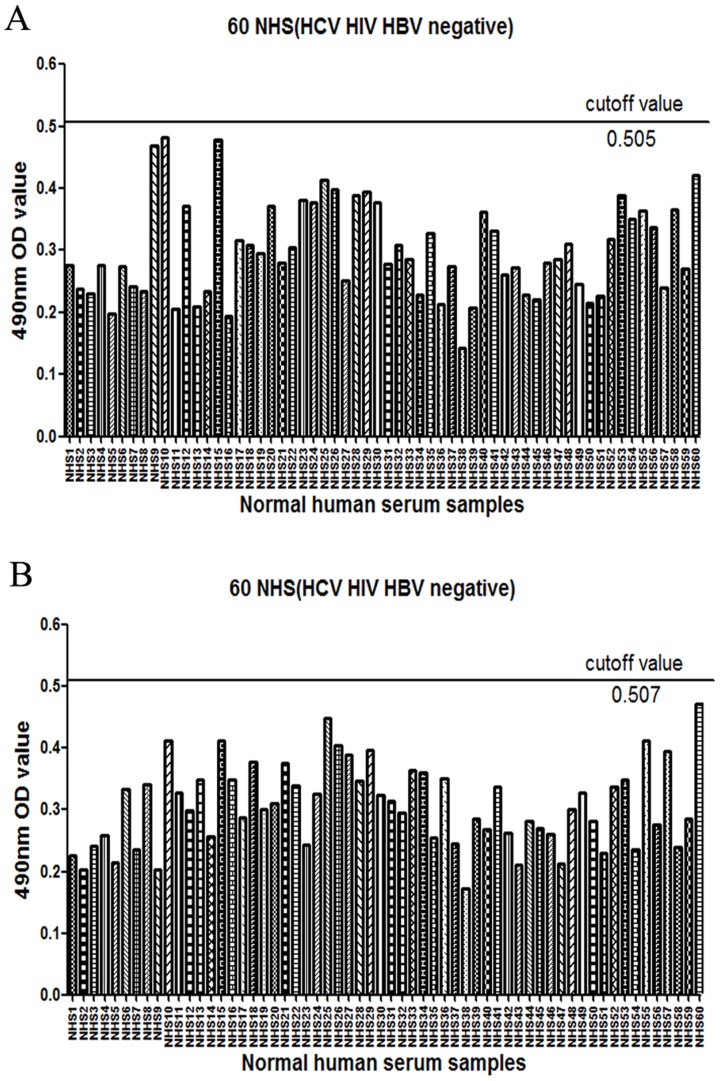
Determination of the cut-off value of 60 NHS samples. The cutoff values of peptide 313–327 and 432–443 for 1/250 dilution were calculated from 60 NHS samples and corresponded to the mean OD values+3SD. (A) The cutoff value of peptide 313–327 was 0.505. (B) The cutoff value of peptide432–443 was 0.507.

Determining the cutoff value for epitope-reactive antibodies was performed as previously describedusing 60 NHS samples [31]. The cutoff value using a standard dilution of 1/250 (HCV, HIV, HBV negative; Fig. 1A, B) was calculated as the mean value+3 SD. Each serum sample was tested in triplicate for each peptide.If the mean value was greater than or equal to the cutoff for a fixed dilution, the sample was defined as either positive. If it was under the cutoff, the sample was considered negative. At least five NHS results were systematically included in each assay, and the cutoff was recalculated for each type of experiment. Positive sera contain 313–327 or 432–443 peptide-reactive antibodies, but negative sera do not contain any of these antibodies.

### Antibody Reactivity to Epitopes 313–327 and 432–443 Differs between Chronically Infected and Spontaneously Cleared Samples

In an effort to better understand the reactivity of the immune system during HCV infection, serum samples from 210 chronically infected individuals (CHC) and 126 individuals who spontaneously clear an HCV infection weretested using theVERSANT HCV Genotype 2.0 Assay (Siemens Healthcare, Belgium). Among CHC samples, 125were genotype 1b, 1 was genotype 1, 67 were genotype 2a, and the remaining 17 patients were infected with a mixture of genotype 1 and 2. The percentages of the total sample size were 59.52%, 0.48%, 31.9% and 8.1% respectively. Other HCV genotypes were not found in theseserum samples.

Within the210 CHCserum samples, 51 had antibodies reactive to epitope 313–327 (24.29%) while antibodies reactive to epitope 432–443 were found in 10 samples(4.76%). In the spontaneousclearance group, 1 (0.79%) sample was positive for antibodies reactive to epitope 313–327 and 16(12.70%) were positive for antibodies reactive to epitope 432–443 (Table 2).Two samples, named CHC65 and CHC123, were displayed positive reactivity against both epitopes. The percentage of samples reactive to epitope 313–327 was significantly higher than those reactive to epitope 432–443(*P*<0.01). Moreover, there was a significantly higher percentage of samples reactive to epitope 313–327 in the CHC group compared to the spontaneous clearance group(*P*<0.01);however thepercentage of samples reactive to epitope 432–443 was more prominent in the spontaneous clearance group than in the CHC group(*P*<0.05).

**Table 2:** Serum reactivity to the 313–327, 432–443 and NS4 peptides differs between infecting HCV genotypes.

Infection type	genotype	313–327 peptide**		432–443 peptide*		NS4 peptide	
		n	No.positive (%)	n	No. positive (%)	n	No. positive (%)
Chronic	1	126	35(27.78)	126	6(4.76)	53	20(37.74)
	2	67	10(14.92)	67	3(4.48)	25	5(20)
	1 mixed 2	17	6(35.29)	17	1(5.88)	6	2(33.33)
Sp. Cleared	ND	126	1(0.79)	126	16(12.7)	30	1(3.33)

** Values for the CHC group are significantlyhigherthan for thespontaneous clearance group (*P*<0.01).

* Values for the spontaneous clearance group are significantly higher than those of the CHC group (*P*<0.05).

### Human Antibody Responses Vary Depending on HCV Genotype

Among the 51 CHC samples that were reactive to epitope313–327,34(66.67%)wereagainst genotype 1b, 1 (1.96%) was against genotype 1, 10 (19.61%) were against genotype 2a, and 6 (11.76%) were against a mixture of genotype 1 and 2. Compared with samples reactive to genotype 2a, samples reactive to genotype 1b were better equipped to produce antibodies reactive to epitope 313–327;however this difference was not statistically significant (*P*>0.05).Ten samplesin the CHC group were reactive to epitope 432–443. Within these positive samples, 6 (60%)were against genotype 1b, 3 (30%) were against genotype2a, and 1 (10%) was against a mixture of infection by genotypes 1and 2; however, no significant difference was found between the reactivity to these genotypes.

**Figure 2:**
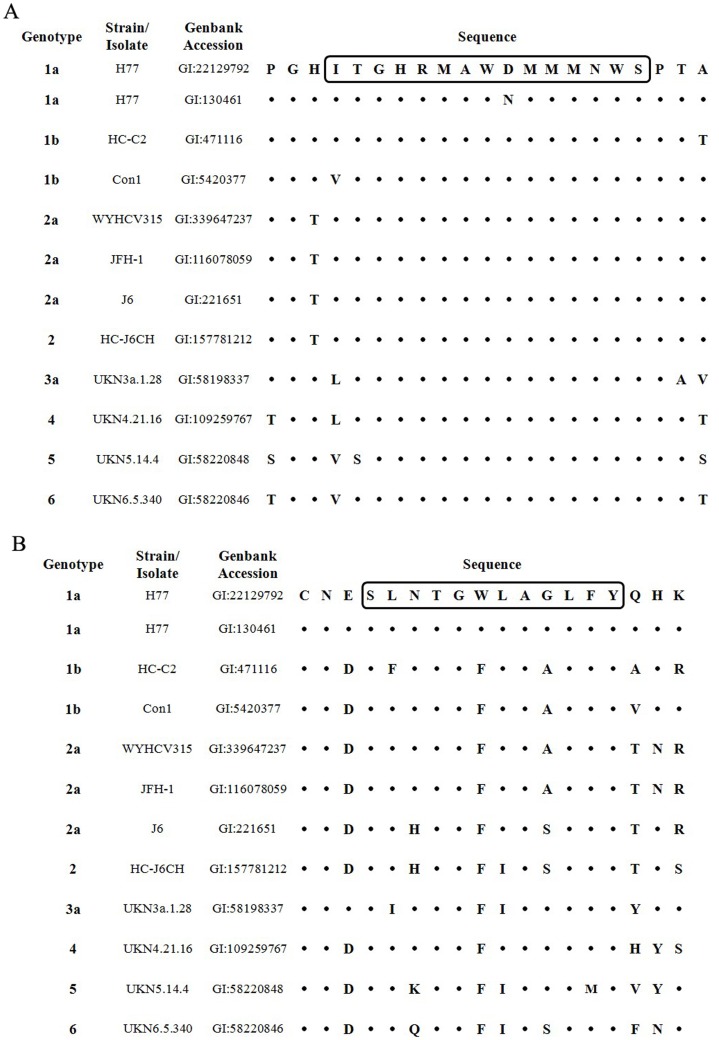
Sequence alignment of 313–327 and 432–443 regions of several HCV strains/isolates. (A) Sequence alignment of region 313–327 from several HCV strains/isolates. (B) Sequence alignment of region 432–443 from several HCV strains/isolates. The boxed sequences were the peptides used for ELISA and peptide blocking assays.

The amino acid sequences of these two regions of several HCV strains that belonged to genotype 1a, 1b, 2a and2 were aligned to show if the variability in these two regions affected ELISA results (Fig. 2A, B). The regioncorresponding to 313–327of H77 (GI: 130461) and Con1 strains harbored the D321N and I313V substitutions, respectively. The corresponding regions of other HCV strains were the same with the peptide used for ELISA and peptide blocking assay (Fig. 2A). The regioncorresponding to 432–443of H77 (GI: 130461), HC-C2, Con1, WYHCV315, JFH-1, J6 and HC-J6CH harboredthe L433F, N434H, W437F, G440A and G440S substitutions, respectively (Fig. 2B). Thus, the differences of the reactivity between genotypes in CHC group might be affected by the variability of these sequences corresponding to epitopes 313–327 and 432–443.

### Antibodyreactivity is Epitope Specific

**Figure 3:**
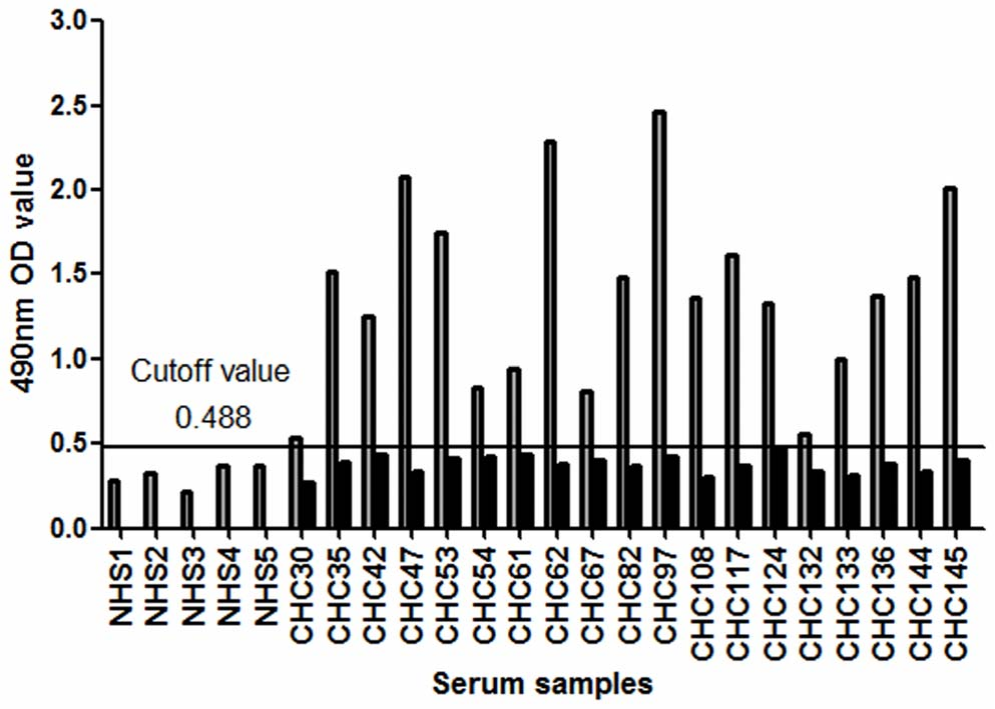
Specificity of epitope-reactive antibody detection tests. Nineteen serum samples reactive to epitope 313–327 from the CHC group were tested in the absence of peptide (no peptide) or in the presence of peptide 313–327. The cutoff value of peptide 313–327 for 1/250 dilution was0.488 (calculated from 5 NHS samples and corresponded to the mean OD values+3SD). Data are represented as the mean values.

Tostrengthen the specificity of the reactivity test, a subset of serum samples was analyzed in the absence of peptide (no peptide).As shown in Fig. 3, 19 CHC samples with reactivity to epitope 313–327 were tested at a 1/250 dilution.All wellswithout peptide were negative,andwells coated with peptide 313–327 were positive. These data suggest that epitope-reactive antibody detection test was highly specific.

### Human Samples Reactive to Epitope 432–443 have Neutralizing Activity Against Multiple Genotypes ofHCVpp

**Figure 4:**
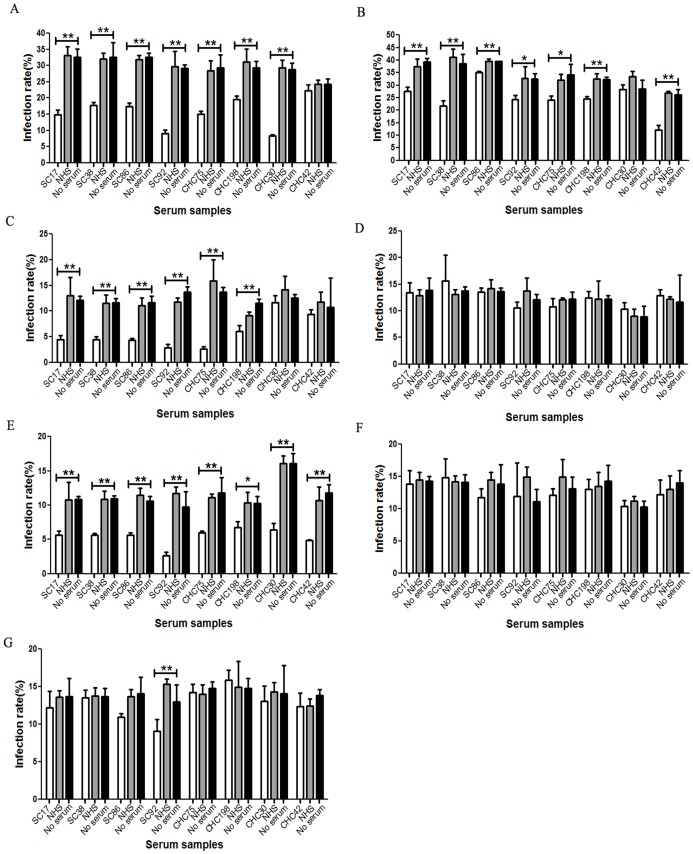
Cross-genotype neutralizing capacity of 432–443 epitope-reactive human serum samples at the dilution 1/100. Six samples reactive to epitope 432–443 have cross-genotype neutralizing activity. Serum was assessed usingneutralizing assays to genotype 1a HCVpp (A), genotype 1b HCVpp (B), genotype 2a HCVpp (C), genotype 3a HCVpp(D), genotype 4 HCVpp (E), genotype 5 HCVpp(F) and genotype 6 HCVpp(G).

In order to test the neutralizing activity of the epitope reactive samples, we tested the ability of these samples to neutralize HCVpp of multiple genotypes. All 26 positive serum samples from spontaneous clearance group and CHC group were tested for their ability to neutralize HCVpp bearing the envelope glycoproteins of genotypes 1a, 1b, 2a, 3a, 4, 5 and 6.Six serum sampleswere found to have cross-genotype neutralizing capacity. Among these samples, SC38 efficiently neutralized genotypes 1a,1b, 2a and 4HCVpp(Fig. 4A, B, C, E).SC17,SC86 and CHC75 efficiently neutralized genotypes 1a, 2a and 4HCVpp and slightly inhibitedthe infection activity of genotype 1b HCVpp(Fig. 4A, C, D, E). SC92 efficiently neutralized genotypes 1a, 2a and 4HCVpp and slightly inhibited the infection activity of genotypes 1b and 6 HCVpp, while CHC198 efficiently neutralized genotype 2a HCVpp and inhibited the infection activity of genotypes1a, 1b and 4HCVpp(Fig. 4A, B, C, E).However, none ofthese 6 samples neutralized genotypes 3a and 5HCVpp(Fig. 4D, F). Re-examination of the sequence data in figure 2, revealed that variability may affect the neutralizing activity of these 6 samples. Compared with genotypes1a, 1b, 2a and 4 HCVpp(Fig. 2B, GI:130461, GI:5420377, GI:116078059, GI:109259767), the L438I substitution occurred only in E2 glycoprotein of genotypes 3a (Fig. 2B, GI:58198337), 5(Fig. 2B, GI:58220848) and 6 (Fig. 2B, GI:58220846). The infection activity of genotypes 3a and 5 HCVpp cannot be inhibited by any of the six sera, while genotype 6 HCVppmay only be slightly inhibited by sample SC92, which suggest that L438 may be critical for the neutralizing activity of 432–443 reactive antibodies. Among these 6 samples, 4came from the spontaneous clearance group and 2 from the CHC group. Other samples could only neutralize homologous genotype HCVpp or could not neutralize any HCVpp genotype mentioned above (data not shown).

### Human Samples Reactive to Epitope 313–327 can Neutralize Homologous Genotypes ofHCVpp

In order to test the functionality of the anti-HCV antibodies generated during HCV infection, 52 samples reactive to epitope 313–327 were also tested for their ability to inhibit HCVpp entry.Among these samples only 1 was from the spontaneous clearance group. We found that 47 of 52 serum samples neutralizedtheir homologous genotype HCVpp (data not shown). Among these 47 samples, CHC30 (genotype 1) and CHC42 (mixed genotype 1 and 2infection) also neutralized genotype 4HCVpp (Fig. 4E). CHC30 neutralized genotypes 1a and 4HCVpp(Fig. 4A, E), while CHC42 neutralized genotypes 1b and 4HCVpp(Fig. 4B, E).However,other genotypes of HCVppwere not neutralized. The sample from the spontaneously clearance group did not neutralize any genotypesofHCVpp used here (data not shown). Together these data suggest that serum samples reactive to epitope 313–327 may not have broad neutralizing activities.

### Neutralizing Activity can be Blocked by Peptide 432–443 but not Peptide 313–327

**Figure 5:**
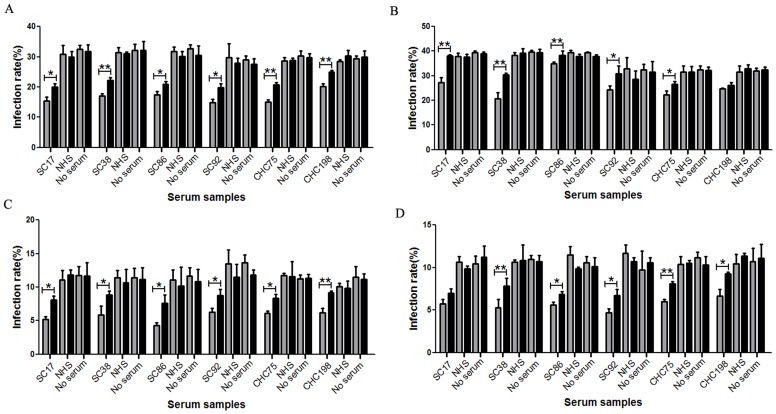
Neutralizing activity of 432–443 epitope-reactive antibodies can be partially inhibited. Exogenous peptide in the presence of sera at a dilution resulting in approximately 50% inhibition of HCVpp infection (dilutions varied between 1∶50 and 1∶600) was used to test inhibition of genotype 1a HCVpp (A), genotype 1b HCVpp (B), genotype 2a HCVpp(C) and genotype 4 HCVpp (D). Shaded bars and filled bars represent control peptide and peptide 432–443, respectively.

**Figure 6:**
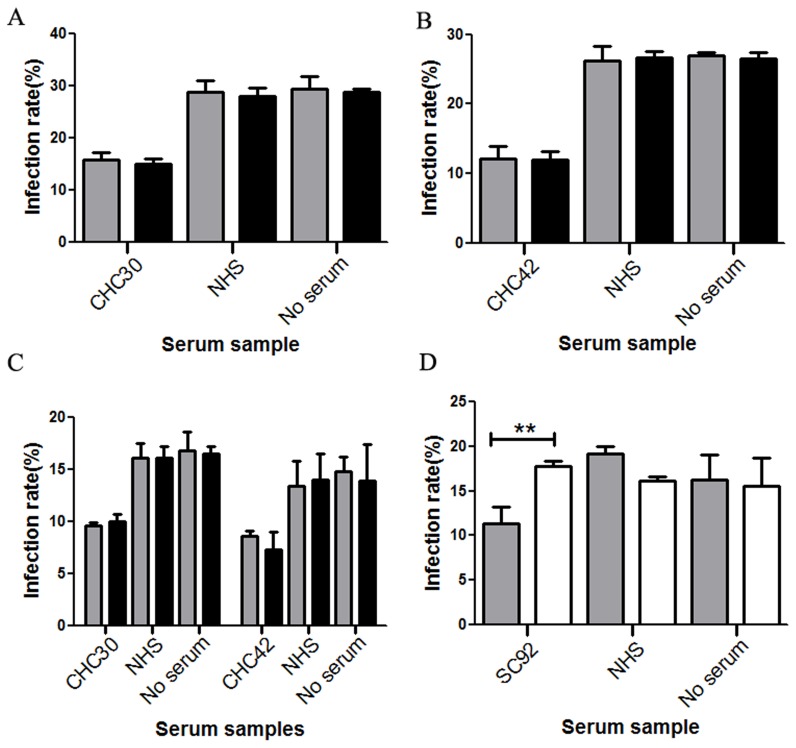
Inhibition of neutralizing activity differs between epitopes 313–327 and 432–443. (A) Neutralizing activity of CHC30was not blocked by peptide 313–327.(B) Neutralizing activity of CHC42 to genotype 1b was not blocked by peptide 313–327. (C) Neutralizing activities ofCHC30 and CHC42 to genotype 4 were not blocked by peptide 313–327. (D) Neutralizing activity of SC92 to genotype 6 was partially blocked by peptide 432–443. Shaded bars, filled bars and open bars represent control peptide, peptide 313–327 and peptide 432–443, respectively.

Todetermine the specificity of the neutralizing activity of antibodies reactive to epitopes 432–443 and 313–327, six samples reactive to epitope 432–443, sample CHC30, and sample CHC42 were used in a competition experiment with saturating amounts of peptides 432–443 and 313–327, respectively.At the IC50 dilution, neutralizing activities ofSC38, SC86, SC92 and CHC75 for genotypes 1a, 1b,2a and 4HCVppwerepartially blocked by the addition of exogenous peptide 432–443(Fig. 5A, B, C, D).The neutralizing activity of SC92 for genotype6HCVppwas alsopartially blocked by peptide 432–443 (Fig. 6D).The neutralizing activity of SC17 for genotypes 1a, 1b and 2a HCVppwerepartially blocked by peptide 432–443 (Fig. 5A, B, C), while this peptide had no effect on the infection activity of genotype 4HCVpp(Fig. 5D). The reactivity of sample CHC198 for genotypes 1a, 2a and 4HCVppwaspartially blocked by peptide 432–443 (Fig. 5A, C, D), but the peptide had no effect on genotype 1bHCVpp(Fig. 5B). Peptide 313–327 had no effect on the neutralizing activityof its binding antibody(Fig. 6A, B, and C). Taken together, these data suggest that antibodies reactive to epitope 432–443 may have cross-genotype neutralizing activities.

### Six432–443 Epitope-reactive Human Antibody-positive Samples have Neutralizing Activity Against Cell Culture-produced HCV Strains of Genotype 2a

**Figure 7:**
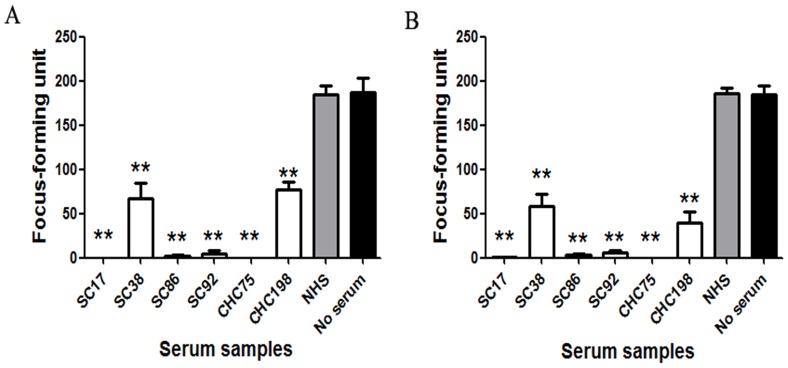
Neutralizing capacity of samples reactive to epitope 432–443 to genotype 2a HCVcc. HCVcc neutralization assays were performed in the presence of diluted 432–443 epitope-reactive samples (1∶100 dilution), and the HCV NS3-postive foci were calculated after 72 hours.(A) Six 432–443 epitope-reactive samples can neutralize JFH-1 virus stocks. (B) Six 432–443 epitope-reactive samples can neutralize J6/JFH-1 virus stocks.

If the neutralization of HCVpp infectivity demonstrated for these sera was biologically relevant, we expected that these sera samples would also neutralize cell-cultured HCV strains. Two cell-cultured strains of genotype 2a, JFH-1 and chimeric J6/JFH-1, were used in the analysis. Therefore, neutralization tests were repeated with HCVcc of these strains. Prior toincubation with Huh7.5 cells, a cell culture supernatant containingHCVcc particles (200 focus-forming units/well) was mixed with each positive sera andcontrol sera at various concentrations.Quantification of foci showed that SC17, SC86, SC92 and CHC75 strongly neutralized JFH-1 and J6/JFH-1 infectivity at the dilution 1/100 (Fig. 7). SC38 and CHC198 alsoefficiently neutralized JFH-1 and J6/JFH-1 at the same dilution (Fig. 7). These data suggest that the neutralization demonstrated by these human samples is biologically relevant.

### Neutralizing Activity of 432–443 Reactive Human Serum Samples can be Partially Blocked by the Corresponding Peptide While 313–327 Reactive Samples cannot be Blocked

**Figure 8:**
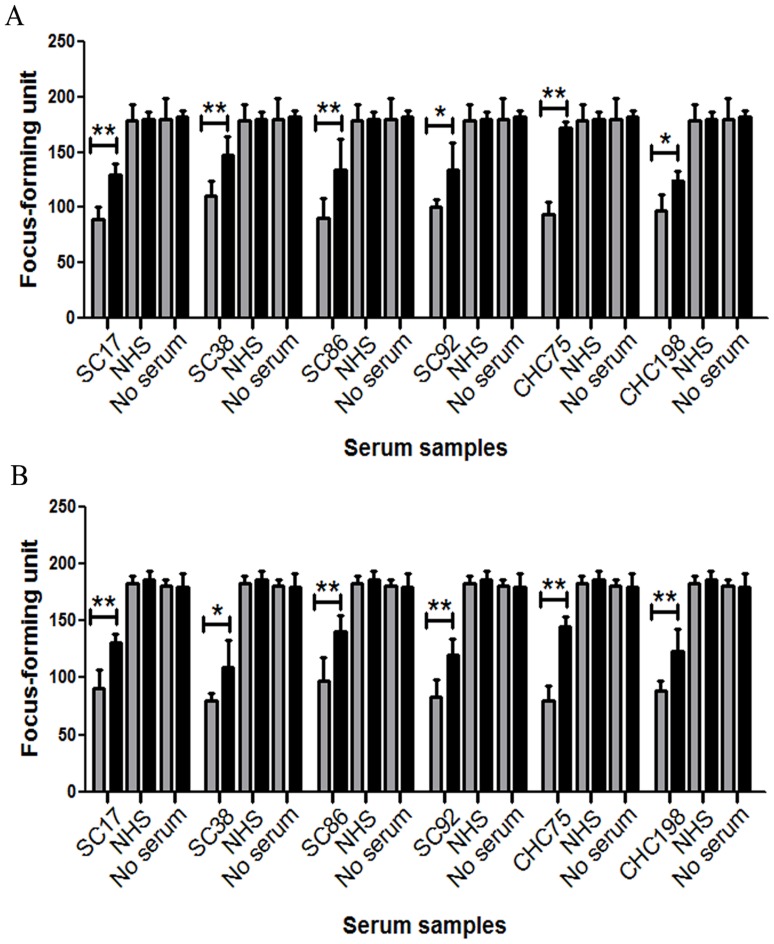
Neutralizing activity of 432–443 epitope-reactive antibodies can be partially blocked by exogenous peptide. Serum samples were cultured in the presence of sera at a dilution resulting in approximately 50% inhibition of HCVcc infection (dilutions varied between 1∶200 and 1∶2000). (A) The neutralizing activity of six 432–443 epitope-reactive samples to JFH-1 virus stocks was partially blocked by peptide 432–443. (B) The neutralizing activity of six 432–443 epitope-reactive samples to J6/JFH-1 virus stocks was partially blocked by peptide 432–443. Shaded bars and filled bars represent control peptide and peptide 432–443, respectively.

Given that six serum samples from two groups containing antibodies recognizing epitope 432–443 are likely to contribute to the sera’s ability to neutralize cell-cultured strains of genotype 2a, we started by assessing the neutralizing potential of these sera. Cell-cultured JFH-1 and chimericJ6/JFH-1 virus (200 focus-forming units/well)were mixed with sera at a dilution that was approximately the IC50 value, in the presence of the 432–443 peptide or an irrelevant negativecontrol peptide, and the resulting HCVcc infectivity was determined (Fig. 8). At the IC50 dilution, neutralizing activities of these six sera were also partially blocked by peptide 432–443. Increasing peptide concentration did not affect the inhibition ofHCVcc neutralization (data not shown). These data suggest that genotype 2a HCVcc stocks can be neutralized by 432–443 epitope-reactive antibodies.

**Figure 9:**
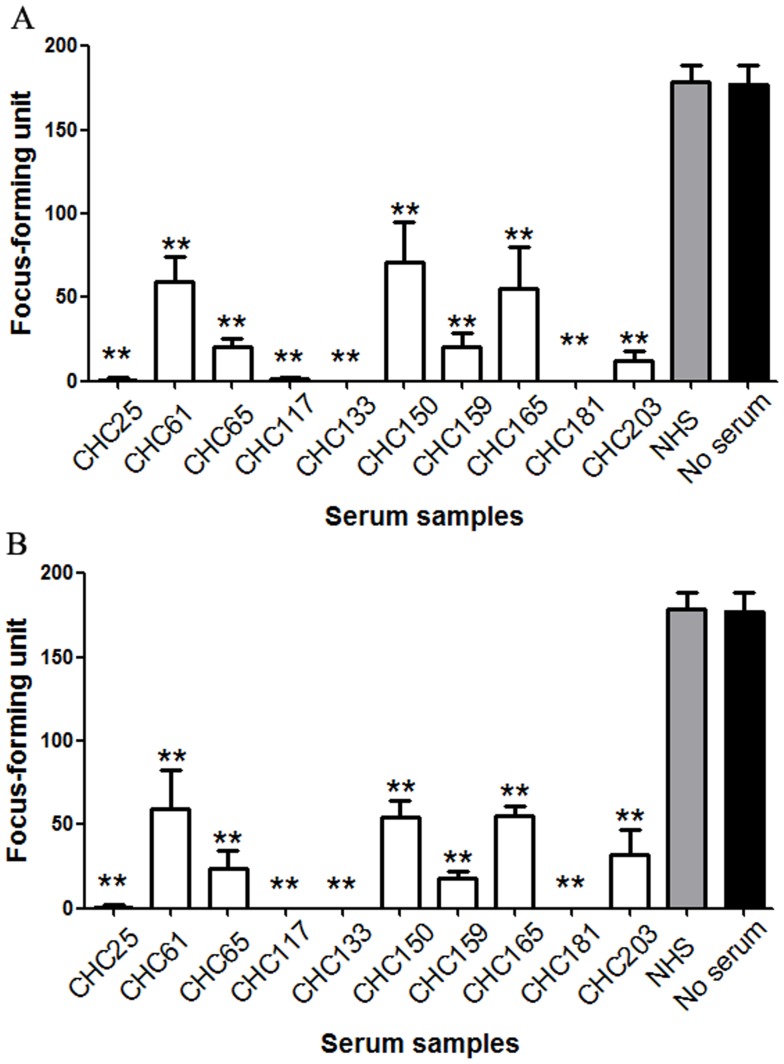
Neutralizing capacity of serum samples reactive to epitope 313–327 to genotype 2a HCVcc. HCVcc neutralization assays were performed in the presence of diluted 313–327 epitope-reactive samples (1∶100 dilution), and the HCV NS3-postive foci were calculated after 72 hours. (A) Ten 313–327 epitope-reactive samples can neutralize JFH-1 virus stocks. (B) Ten 313–327 epitope-reactive samples can neutralize J6/JFH-1 virus stocks.

**Figure 10:**
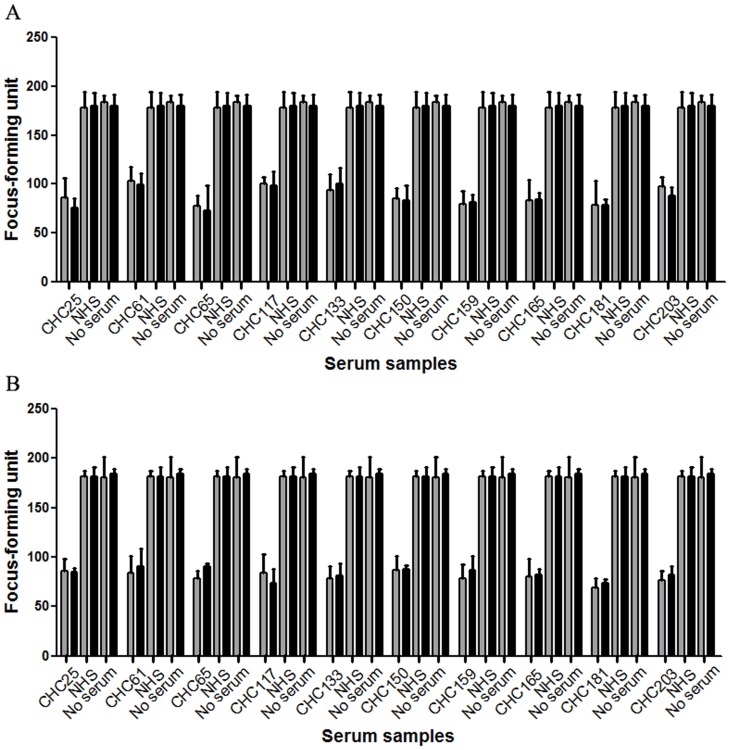
Neutralizing activity of serum samples reactive to epitope 313–327 cannot be blocked by corresponding exogenous peptide. Serum samples were cultured in the presence of sera at a dilution resulting in approximately 50% inhibition of HCVcc infection (dilutions varied between 1∶200 and 1∶1600). (A) The neutralizing activity of ten samples reactive to epitope 313–327to JFH-1 virus stockscannot be blocked by peptide 313–327. (B) The neutralizing activity of ten samples reactive to epitope 313–327to J6/JFH-1 virus stockscannot be blocked by peptide 313–327. Shaded bars and filled bars represent control peptide and peptide 313–327, respectively.

Fifty-two samples reactive to epitope 313–327 were also tested for an ability to neutralize genotype 2a HCVcc (JFH-1 and chimericJ6/JFH-1). Prior toincubation with Huh7.5 cells, a cell culture supernatant containingHCVcc particles (200 focus-forming units/well) was mixed with each positive sera andcontrol sera at various concentrations. Ten serum samples named CHC61, CHC65, CHC117, CHC150, CHC159, CHC165, CHC181, CHC203 (genotype 2), CHC25 and CHC133 (mixed genotype 1 and 2infection) had neutralizing activity (Fig. 9). One sample came from the spontaneous clearance group, however, it did not neutralize genotype 2aHCVcc (data not shown).We nextassessed the contribution of antibodies reactive to epitope 313–327 to the neutralizing potential of these sera. Cell-cultured JFH-1 and chimericJ6/JFH-1 virus (200 focus-forming units/well)were mixed with sera at a dilution that was approximately the IC50 value (dilutions varied between 1∶200 and 1∶1600), in the presence of the 313–327 peptide or an irrelevant negativecontrol peptide, and the resulting HCVcc infectivity was determined (Fig. 10). At the IC50 dilution, neutralizing activities of these 10 sera could not be blocked by addition of exogenous peptide 313–327. These ten serum samples can also neutralize genotype 2a HCVpp, while neutralizing activities cannot be blocked by corresponding peptide (data not shown).

## Discussion

It has been reported that MAbs againstHCV may have the potential to neutralize viral entry [24], [33]. The protective epitopes targeted by these MAbs have been mapped to the regionsencompassing amino acids 313–327 and 432–443. In this study, we synthesized these two peptides and tested the reactivity of serum samples from 336 patients, 210 of whichwere from Chronic Hepatitis C (CHC) patients infected with diverse HCV genotypes.Our data revealed that sera reactive to epitope 313–327 could only neutralize homologous genotype HCVpp and/or HCVcc. Two exceptions being samples CHC30 and CHC42,that could also neutralize genotype 4HCVpp.Neutralizing activity was not blocked using exogenous peptide 313–327 in any of the samples tested.This data suggests that 313–327 epitope-reactive antibodies in these samples may not have neutralizing activities.

One study has reported that serum antibodies reactive to epitope 313–327 can strongly neutralize HCVpp bearing the envelope glycoproteins of genotypes 1a, 1b, 4, 5 and 6.In addition, the antibodies can also neutralize HCVpp bearing the envelope glycoproteins of genotype 2a, but to a lesser extent. Genotype 3a was not neutralized [24]. The data reported here draws different conclusions. First, in their study the authors argue that region 313–327 contains multiple but mostly conformational epitopes; however the antibodies recognizingconformational epitopes might not be detected by assays based on synthetic peptidesused in our study. Second, we hypothesize that these antibodies recognize overlapping but distinct epitopes,and that the protective epitopes within peptide 313–327 might not be recognized in samples collected from individuals naturallyinfected with HCV.Therefore, the neutralizing activities reported byMeunier et al. are quite different [24]. Finally, studies have shown that binding of non-neutralizing antibodiesto a virus can interfere with neutralizing antibody capability [33], [41].In our study, polyclonal sera containing all the antibodies were used, and non-neutralizing antibodies may haveinterfered with the activity of neutralizing antibodies, and may beanother reason for the narrow reactivity of serum reactive to epitope 313–327.

Differences in reactivity were also observed to epitope 432–443. Our data revealed that 6 serum samples reactive to epitope 432–443 could effectively neutralize genotypes 1a,2a, 4HCVpp; genotype 2a HCVcc;and weakly neutralize or inhibit the infection activity of genotypes1band/or 6 HCVpp. However, no samples were able to neutralize genotypes 3a and 5HCVpp. This may be due to the fact that amino acid substitution in some sites of the region432–443 of these two genotypes HCVpp may provide resistance to the sera’s neutralizing activity.

We also examined the specificity of the neutralizing antibodies. Among twenty-six 432–443 epitope-reactive human antibody-positive samples,six samples (SC17, SC38, SC86, SC92, CHC75 and CHC198) have neutralizing activity against multiple genotypes ofHCVpp and genotype 2a HCVcc.Neutralizing activities of samples SC38, SC86, SC92 and CHC75were partiallyblocked by addition of exogenous peptide 432–443.However,the neutralizing activity of sample SC17 for genotype 4 and sample CHC198 for genotype 1b HCVppwere notinhibited by addition of this peptide. Thus, these data suggest that antibodies reactive to epitope 432–443 may have the ability to prevent infection by genotypes 1a, 1b, 2a and 4HCVpp and genotype 2a HCVcc,which is consistent with previous studies [32], [33].However,neutralizingactivities of 432–443 epitope-reactive antibodies were different and were not absolutely blocked by exogenouspeptide. There are several, non-mutually exclusive, possibilities for this observation. First, antibodies may recognize overlapping but distinct epitopes. In our study epitope samples reactive to epitope 432–443 had cross-genotype neutralizing activity, which is similar to antibodies targeting the region encompassing amino acids 434–446 reported by Tarr et al.,in their study the authors argue thatneutralizing activities of 434–446 epitope-reactive antibodies are different [29].Consistent with this idea, our data revealed that serum samples reactive to epitope 432–443 have different neutralizing activities.Thus, amino acid region 432–443 may contain multiple epitopes,and antibodies directed to the peptide may recognize overlapping yet distinct epitopes.Second,antibodies targeting other regions may also be involved.In our study, some samples were able to neutralize other genotypes of HCVpp, butwere not inhibited by exogenous peptide 432–443. These data suggestthat other anti-HCV antibodies may be responsible for the observed neutralizing activity.Finally, studies have shown that binding of non-neutralizing antibodiesto a virus can interfere with neutralizing antibody capability [33], [41].In our study, polyclonal sera containing all the antibodies were used, and non-neutralizing antibodies may haveinterfered with the activity of neutralizing antibodies.

Because the serum used in this study included both chronic infection and spontaneously cleared samples, we were able to broadly assess the immunogenicity of epitopes 313–327 and 432–443 in natural HCV infection. The prevalence of serum reactive to epitope 313–327 in the CHC and spontaneously cleared group was 24.29% and 0.79%, respectively. On the other hand, serum samples reactive to epitope 432–443 for these two groups was4.76% and 12.70%, respectively. An immunogenic epitope (KPAIIPDREVLYREFDEM; aa 1691–1708) [36]in the NS4 protein-binding antibodies and a control epitope peptide corresponding to a sero-reactive region of the rabies virus glycoprotein (VNLHDFRSDEIE) [37]werealso tested to ensure that the peptide capture assay used here was reliable.Reactivity to peptide 1691–1708 was observed in 32.14% of the 84 CHC serum samples tested here, similar to results reported by Tarr etal [9], but slight lower than earlier findings [36]. No serum sample was reactive to the rabies virus control peptide. The percentage of positive serum samples against epitope 313–327in the CHC group was 24.29%, showing that this epitope has potent immunogenicity. However,these antibodies did not neutralize any genotypesof HCVpp, suggesting thatthey were different from MAbs previously reported [24]. If this epitope is used to developa vaccine in the future,the immunogen must be designed to elicita neutralizing antibody response and to enhance immunogenicity.

Our data do, however, provide evidence that antibodies reactive to epitope 432–443 may be generated in humans by immunization, and some of these antibodies have cross-genotype neutralizing capability.Therefore, epitope 432–443 may be useful in the development of antibody therapies. One study reported that HCV1, a cross-genotype neutralizing antibody, may be an effective therapy for the prevention of graft infection in HCV-infected patients undergoing livertransplantation [33]. However, the mutation of N415K/D and N417S of E2 of HCV virus conferred resistance to HCV1 neutralization, while epitope 432–443-specific antibody, 96–2, could potently neutralizeH77N417S/Q444R–HCVpp. This report, together with the data presented here, suggest that antibodies reactive to epitope 432–443 may be another potentiallyeffective therapyfor the prevention of graft infection. Our data confirm that 432–443-reactive antibodies have different neutralizing activities, which implies that generating antibodies specific for the 432–443 epitope requires alternative approaches for developing an appropriate immunogen. Above all, this study identifies the neutralizing ability of endogenous anti-HCV antibodies and warrants the exploration of antibodies reactive to epitope 432–443as sources for future antibody therapies.
